# Ellagic Acid Prevents Dopamine Neuron Degeneration from Oxidative Stress and Neuroinflammation in MPTP Model of Parkinson’s Disease

**DOI:** 10.3390/biom10111519

**Published:** 2020-11-06

**Authors:** Mustafa T. Ardah, Greeshma Bharathan, Tohru Kitada, M. Emdadul Haque

**Affiliations:** 1 Department of Biochemistry, College of Medicine and Health Sciences, UAEU, Al Ain, UAE; Mustafa_Ardah@uaeu.ac.ae (M.T.A.); greeshma_b@uaeu.ac.ae (G.B.); 2 Otawa-Kagaku Service, Parkinson’s Clinic and Research, Kamakura 247-0061, Japan; tohrukitada@gmail.com

**Keywords:** ellagic acid, neurodegeneration, neurotoxicity, Parkinson’s disease

## Abstract

Parkinson’s disease (PD) is one of the most common neurodegenerative diseases and is characterized by progressive dopaminergic neurodegeneration in the substantia nigra pars compacta area. In the present study, treatment of EA for 1 week at a dose of 10 mg/kg body weight prior to MPTP (25 mg/kg body weight) was carried out. MPTP administration caused oxidative stress, as evidenced by decreased activities of superoxide dismutase and catalase, and the depletion of reduced glutathione with a concomitant rise in the lipid peroxidation product, malondialdehyde. It also significantly increased the pro-inflammatory cytokines and elevated the inflammatory mediators like cyclooxygenase-2 (COX-2) and inducible nitric oxide synthase (iNOS) in the striatum. Immunohistochemical analysis revealed a loss of dopamine neurons in the SNc area and a decrease in dopamine transporter in the striatum following MPTP administration. However, treatment with EA prior to MPTP injection significantly rescued the dopaminergic neurons and dopamine transporter. EA treatment further restored antioxidant enzymes, prevented the depletion of glutathione and inhibited lipid peroxidation, in addition to the attenuation of pro-inflammatory cytokines. EA also reduced the levels of COX-2 and iNOS. The findings of the present study demonstrate that EA protects against MPTP-induced PD and the observed neuroprotective effects can be attributed to its potent antioxidant and anti-inflammatory properties.

## 1. Introduction

Parkinson’s disease (PD) is a progressive neurodegenerative disorder associated with selective loss of nigrostriatal dopamine (DA) neurons and the presence of intraneuronal cytoplasmic inclusions known as Lewy bodies. The molecular mechanisms of such selective loss of DA neurons in PD are not fully understood, but excessive free-radical generation, inflammation, and mitochondrial injury—particularly inhibition of mitochondrial complex-1, dysfunction of the ubiquitin proteasome, and autophagy—are believed to be involved. The exact cause of PD is not clearly understood; available scientific evidence suggests that both genetic and environmental risk factors are associated in the etiology of PD. In the laboratory, exposure of several toxicants, such as rotenone, paraquat, and 1-methyl-4-phenyl 1,2,3,6 tetrahydropyridine (MPTP), causes mitochondrial defects and leads to dopamine neuron loss in the SNc neurons [1,2,3]. Among the various dopaminergic neurotoxins used to create a PD animal model, MPTP has received much attention due to its capability to produce clinical features of PD in humans and monkeys. In fact, it was first identified among drug abusers who consumed narcotics drugs contaminated with MPTP. MPTP is highly lipophilic and readily crosses the blood-brain barrier to enter brain cells—astrocytes. In astrocytes, MPTP is metabolized into its toxic metabolite, chemically known as 1-methyl-4-phenylpyridinium (MPP+), which then freely enters the dopamine neurons using dopamine transporter (DAT). After entering the cells, it causes the death of dopamine neurons by inhibiting complex I of the mitochondrial electron transport chain and produces Parkinsonism in experimental animals like rodents and primates. It also displaces the neurotransmitter, dopamine molecule, from the synaptic vesicle and enhances dopamine autoxidation in the cytosol to generate reactive oxygen species that are also detrimental to the neurons. As MPTP causes the selective demise on SNc neurons, it has been widely used to develop animal models of PD to test new therapies or potential drugs. It is noteworthy to mention that an MPTP-induced PD model also offers a valuable animal model for studying molecular mechanisms of dopaminergic neurodegeneration. However, MPTP exposure in mice does not produce LB-like protein aggregates and is not associated with α-syn aggregation.

Numerous studies have shown that a number of plants products, medicinal plant extracts, and phytochemicals, including polyphenols, terpenes, and flavonoids, exert significant antioxidant and anti-inflammatory actions and are effective against several chronic neurodegenerative diseases including PD [4,5,6]. Recently, one of the polyphenol compounds, ellagic acid (EA), a metabolite of ellagitannin, has earned much attention due to its many physiological and pharmacological activities. Ellagic acid is present in many fruits such as pomegranates, persimmons, and raspberries, among others. Structurally, it has four hydroxyl groups that are responsible for its antioxidant property. The medicinal and pharmacological properties of ellagic acid have been reviewed and discussed elsewhere [7]. Several lines of evidence suggest that ellagic acid has many therapeutic benefits such as being anti-inflammatory, neuroprotective, hepatoprotective, antidiabetic, anticancer, and cardiovascular disease fighting agent [8,9,10,11,12,13]. It has been demonstrated that ellagic acid prevents rotenone-induced generation of S-nitrosylation of protein disulfide isomerase (SNO-PDI), a marker of Parkinson’s disease in the cellular model [14]. EA has been shown to prevent dopamine neurons from rotenone-induced neurotoxicity through activating Nrf2 signaling cascade [15]. Rotenone inhibits the complex I of mitochondrial electron transport chain in a nonspecific manner and causes cellular demise [16]. Several studies also reported that EA improved motor defects and prevented neuroinflammation against 6-OHDA in a model of PD [17,18,19]. However, they did not provide comprehensive data related to dopamine neuron integrity in the SNc area as a pathological hallmark of PD. Thus, it is not clear whether the observed effect of EA in this model was due to the preservation of DA neurons or through another mechanism. Since available studies indicating the improvement in motor parameters and in vitro data suggest its potential benefit in PD treatment, EA attracted our attention to investigate its neuroprotective effect in a more reliable and classic in vivo animal model that mimics PD in humans.

EA is well known for scavenging free radicals, similar to antioxidant vitamins like vitamins C and E. Besides its antioxidant function, ellagic acid has been shown to exert potent anti-inflammatory activities by inhibiting pro-inflammatory cytokines. However, the antioxidant and anti-inflammatory activities of ellagic acid in a PD brain remain poorly understood. The oxidative stress and inflammation together play critical role in causing selective loss of dopaminergic neurons in the SNc and a reduction in dopamine transporter in the striatum of the PD brain. This ultimately leads to a decrease in dopamine levels in the striatum and produces motor symptoms in PD [20]. Thus, the aim of the present study was to evaluate the neuroprotective potential of EA in an MPTP-induced PD model of mice. We performed the experiment to assess the status of antioxidant enzymes, proinflammatory cytokines, inflammatory mediators, and dopaminergic neurons in an MPTP-induced mice model of PD. Our findings suggest that the neuroprotective action of EA is mediated by its antioxidant and anti-inflammatory activities. The preservation of dopamine neurons and dopamine transporter in the nigrostriatal pathway further confirmed the integrated protective role of EA against DA neurodegeneration in the MPTP model.

## 2. Materials and Methods

### 2.1. Experimental Animals

Two- to three-months old male C57BL6c mice (25–30 g body weight) were received from the animal research facility of the College of Medicine and Health Sciences, United Arab Emirates University, UAE. The mice were kept in a cage for several days to acclimatize to the experimental conditions and were used in the current study. The animals were maintained under standard laboratory conditions of light and dark cycle. The animals had access to commercially available rodent food and water ad libitum. All experiments were performed between 9:00 a.m. and 5:00 p.m. The experimental protocol for animal experimentation was approved by the Animal Ethics Committee of the College of Medicine and Health Sciences, United Arab Emirates University, UAE (Approval number: ERA_2020_6166).

### 2.2. Experimental Design

Mice were injected with MPTP once daily for 5 consecutive days through the intraperitoneal route (25 mg/kg body weight measured as free base; MPTP-HCL; Sigma-Aldrich, St. Louis, MO, USA). Each bottle containing 10 mg MPTP was first dissolved in sterile normal saline to obtain the desired concentration for injection as mentioned above. The dose of MPTP used in the current study to induce Parkinsonism in mice was adopted from our previous report [21]. In order to test the neuroprotective efficacy, EA was first dissolved in a minimal volume of ethanol and was then diluted in sterile normal saline to achieve the desired concentration for injection. We injected EA through the intraperitoneal (IP) route at a dose of 10 mg/kg body weight once daily, 60 min prior to each MPTP administration and continued this for 1 week. We injected EA 60 min prior to MPTP to eliminate any possible reaction between EA and MPTP. The dose of EA in the current study was selected based on a dose-dependent study (5, 10, and 20 mg/kg/body weight) in a different set of experiments that did not show any adverse effect or toxicity in vivo (unpublished data). The animals in the control groups received the same amount of vehicle without EA and saline (Group I) or with EA and saline (Group II). To carry out the experiment, we divided the sample into four groups, each group consisting of 6–8 mice. The groups were organized as follows:

Group I: Vehicle and saline-injected control group (V + Sal)
Group II: Ellagic acid and saline-injected group (EA + Sal)
Group III: Vehicle-treated and MPTP-injected group (V + MPTP)
Group IV: Ellagic acid-treated and MPTP-injected group (EA + MPTP)

### 2.3. Tissue Preparation for Biochemical Studies

Pentobarbital (40 mg/kg body weight) was used at the end of the experiments to anaesthetize the animal, and cardiac perfusion was conducted using 0.01 M phosphate-buffered saline (PBS), pH 7.4, to clear the blood from the brain. The brains were quickly removed and placed on an ice-cold brain matrix. The striatum region was dissected precisely using a blade and was immediately frozen in liquid nitrogen for further biochemical assays.

### 2.4. Biochemical Studies

For biochemical assays, we used KCl buffer (Tris-HCl 10 mM, NaCl 140 mM, KCl 300 mM, EDTA 1 mM, Triton X-100 0.5%, and 1x PPI) to homogenize the striatum tissue sample from each group as reported previously [21]. The tissue homogenates of the striatum were then centrifuged for 20 min at 4 °C at a speed of 14,000× *g*. The total protein in the cleared supernatant was collected and stored at −80 °C for further analysis. A small aliquot of supernatant was used to estimate protein by standard BCA method.

#### 2.4.1. Estimation of Lipid Peroxidation

A malondialdehyde (MDA) Assay Kit (North West Life science, Vancouver, WA, USA) was used to measure the amount of lipid peroxidation as a marker of oxidative stress in the samples following the manufacturer’s instruction and as described before [21]. Briefly, 1 mg total protein of the sample or 125 µL of standard was incubated in the presence of acid reagent (125 µL) and thiobarbituric acid (125 µL) and vortex at maximum speed. Then, the samples were incubated for 60 min at 60 °C, and centrifugation was carried out at 10,000× *g* for 2–3 min. The reaction sample was then transferred to a 96-well clear plate (Thermo Scientific, Waltham, MA, USA) (150 µL/well in duplicate) and the spectra were recorded at 512 nm. Results were expressed as µM MDA/mg protein.

#### 2.4.2. Reduced Glutathione (GSH) Estimation

To estimate GSH, a GSH Assay Kit (Sigma-Aldrich, St. Louis, MO, USA) was used following manufacturer’s instructions as described before [21]. Briefly, 50 μmol/GSH standard solution was prepared by mixing 100 μL of 200 μmol/L GSH standard solution and 300 μL of 0.5% 5-sulfosalicylic acid (SSA). Using this solution, the following GSH standard was prepared by serial dilution using 0.5% SSA solution: 50.0, 25.0, 12.5, 6.25, 3.13, 1.57, and 0 μmol/L. Triplicate samples containing 40 μL of GSH standard solution or samples were added to 96-well plate and the absorbance was measured at 405 nm using plate reader. Results were expressed as µM GSH/mg protein.

#### 2.4.3. Antioxidant Enzymes Activities Estimation

The activities of superoxide dismutase (SOD) and catalase (CAT) were measured using commercial kits (Cayman Chemicals Company, Ann Arbor, MI, USA) following the manufacturer’s instructions and as described before [21]. Briefly, for catalase assay, a standard solution of formaldehyde was prepared as 0, 5, 15, 30, 45, 60, and 75 µM and then added to 20 µL of the sample or positive control followed by adding 30 µL methanol to each well. To initiate the reaction, 20 μL of diluted hydrogen peroxide was added and samples were incubated for 20 min at room temperature. To terminate the reaction, 30 μL of potassium hydroxide was added to each well followed by 30 μL of Catalase Purpald (Chromogen). The absorbance at 540 nm was read after adding 10 μL of catalase potassium periodate. For SOD measurement, 10 µL of the samples or standard were added to a 96-well plate and the reaction was initiated by adding 20 µL of xanthine oxidase to each well and incubating for 30 min at room temperature after shaking the plate for a few seconds. Absorbance was read using the microplate reader at 450 nm. The CAT activity was expressed as nmol/min/mg protein, and the SOD activity was expressed as U/mg protein.

#### 2.4.4. Pro-Inflammatory Cytokines Estimation

The level of pro-inflammatory cytokines such as interleukin-1β (IL-1β), interleukin-6 (IL-6), and tumor necrosis factor-alpha (TNF-α) was estimated using the commercially available kits procured from R&D systems (Minneapolis, MN, USA). As per the manufacturer’s instructions, the 96-well plate was coated by the diluted capture antibody (100 µL) for overnight at RT. The plate was blocked with reagent diluent (1% bovine serum albumin in PBS) 300 µL for 1 h, followed by washing with wash buffer (0.05% tween 20 in 0.01 M PBS at pH 7.4). Then, 100 µL of samples or standard was added and incubated for 2 h. After washing, the detection antibody (100 µL) was then added and the plate was further incubated for 2 h at RT. One hundred microliters of diluted (1:200) streptavidin horseradish peroxidase was then added and incubated for 20 min. The substrate solution (100 µL) was added and incubated for another 20 min. To stop the reaction, a stop solution (2N H2SO4; 50 µL) was added to the plate and samples were mixed by gentle tapping. Absorbance at 450 nm was recorded immediately using a microplate reader, and the results were expressed as pg/mg protein.

#### 2.4.5. Western Blot Analysis of COX-2 and iNOS

To quantify the levels of COX-2 and iNOS, the striatum tissue lysate was also used in this assay. Samples containing 20 μg of protein were separated in 15% SDS-polyacrylamide gel, and then the separated proteins were transferred onto PVDF membrane and incubated with specific primary rabbit polyclonal antibody against COX-2 (1:1000) and iNOS (1:500) for overnight at 4 °C. After several washings, the membrane was probed with horseradish peroxidase (HRP)-conjugated secondary antibody for 1 h at RT. The immunoreactive bands were visualized using a West Pico chemiluminescence kit following manufacturer’s guidelines (Thermo Scientific, Rockford, IL, USA). For loading control, the membranes were stripped and reprobed with GAPDH (1:1000; rabbit monoclonal, Cell Signaling Technology, Beverly, MA, USA). Samples from three animals from each group were used for Western blot analysis and further statistical analysis. Bands quantification was carried out using Image J software.

### 2.5. Immunostaining and Assessment of Tyrosine Hydroxylase (TH) in the SNc and Dopamine Transporter (DAT) in the Striatum

Mice were sacrificed 2 weeks after the start of MPTP and brains were collected after perfusion. The brains were processed by sectioning and used for TH and dopamine transporter (DAT) immunohistochemical analysis. To assess and evaluate the integrity of TH neurons, we employed optical fractionation using a Stereo Investigator (version 2017), as described previously [21]. In brief, mouse brain was sectioned at a thickness of 40 µm within the rostral and caudal limits covering the SNc area (from −2.54 to −3.88 mm of bregma) and was collected. For each brain, a total of seven coronal sections containing every sixth section from the SNc area were pulled together for immunohistochemical analysis and used for counting the TH neurons number. In brief, serial sections of mouse brain from each mouse covering the TH neurons in the SNc area were washed twice with 0.01 M phosphate buffered saline (PBS), pH 7.4, and were then incubated with blocking reagent (10% normal goat serum in PBS 0.3% Triton-X 100) for 1 h. Further, the sections were incubated with the primary polyclonal rabbit antibody against TH (1:500) for 2 days at 4 °C. Brain sections were then washed with PBS for three times and incubated further with a secondary antibody overnight at 4 °C. The immunoreactivity of the antibody was visualized by avidin-biotin complex peroxidase reaction of DAB as described in [21]. After immunostaining, the brain sections were mounted on a slide and defatted and cover slipped using mounting medium. The thickness of the brain section was measured with a *z*-axis microcreator according to the manufacturer’s instructions. Sections were analyzed using an objective of 63×. The total number of TH-positive neurons was determined using the optical fractionator.

The decrease in striatal nerve fibers was evaluated by measuring the optical density of immuno-stained dopaminergic fibers in the striatum area (adjacent to 0.3 mm of bregma) using Image J software (NIH, Bethesda, MD, USA). Briefly, brain sections covering the striatum area were washed twice with 0.01 M phosphate buffered saline (PBS) pH 7.4 and then incubated with blocking reagent (10% normal goat serum in PBS 0.3% Triton-X 100) for 1 h. Further, the sections were incubated with the primary polyclonal rat antibody against DAT (1:500) overnight at 4 °C followed with a secondary antibody overnight at 4 °C. The immunoreactivity of the antibody was visualized by avidin-biotin complex peroxidase reaction of DAB. The images of DAT immunostained striatum were captured, keeping microscope settings the same for all sections and for optical density analysis. The optical density of the dopamine transporter at three different fields of each section with similar anatomical areas within the striatum was measured for each mouse and an average of the three areas was calculated and is represented as a percentage with reference to the control group (Vehicle + Saline). The optical density of the overlying cortex was taken as a background measure and subtracted from the value generated from the striatum. The counting of TH-positive neurons and optical density of the DAT was carried out by an investigator blind to the experimental groups.

### 2.6. Immunofluorescence Staining for Astrocyte (GFAP) and Microglia (Iba-1)

Immunofluorescence staining was performed with the brain section containing the striatum area to examine the expression and morphology related to activation of astrocyte (GFAP) and microglia (Iba1). Briefly, 40 µm coronal brain sections containing striatum were incubated with proteinase K (5 µg/µL) for 30 min at 25 °C to allow the penetration of the antibody. After washing with PBS, the sections were incubated with blocking reagent (10% normal goat serum in PBS containing 0.3% Triton-X 100). The sections were then incubated with the primary antibodies such as anti-GFAP (1:1000) and anti-Iba-1 (1:1000) overnight at 4 °C. The sections were then briefly washed three times with PBS and incubated for 1 h with a fluorescent secondary antibody (Alexa Flour 488 goat antirabbit) at room temperature. Sections were then washed thoroughly and mounted on a slide. The sections were then cover slipped using Fluoroshield mounting media (Sigma-Aldrich, St. Louis, MO, USA). Images were taken randomly, selecting 3–5 files per sample with a Leica fluorescent microscope at 20× (Leica DFC 3000G). Images were used to count activated astrocyte (GFAP) and microglia (Iba-1) per field using NIH image J software.

### 2.7. Protein Estimation

The total soluble protein was determined using the Pierce BCA Protein Assay Kit (Thermo Scientific, Rockford, IL, USA) following the manufacturer’s instructions.

### 2.8. Statistical Analyses

Data were expressed as the mean value ± SEM. The data for all studies were analyzed using GraphPad software (unpaired Student’s *t*-test), and asterisks represent significant differences: * *p* < 0.05, ** *p* < 0.01, and *** *p* < 0.001.

## 3. Results

### 3.1. EA Prevents MPTP-Induced Dopaminergic Neurodegeneration in SNc and Dopamine Transporter in Striatum

**Figure 1:**
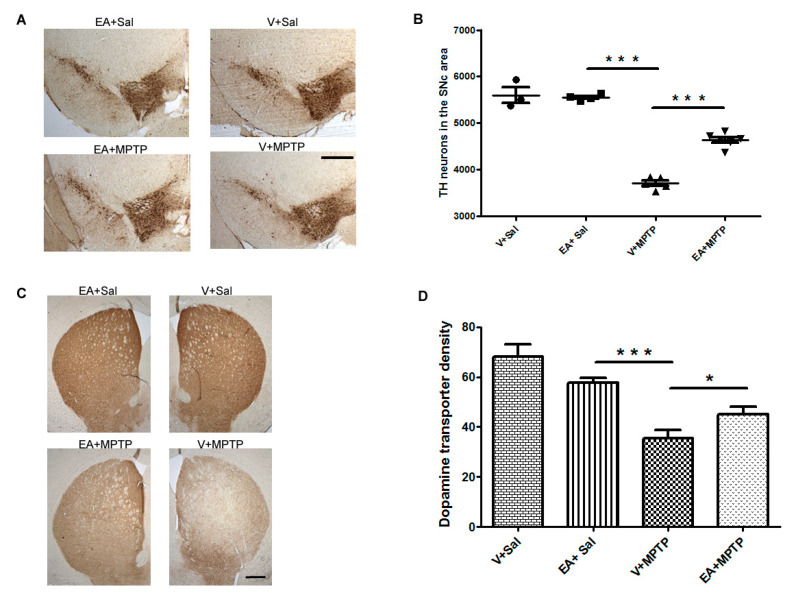
Immunostaining of tyrosine hydroxylase immune-positive (TH^+^) neurons to quantify the number of dopaminergic (DA) neurons in the substantia nigra (SNc) and dopamine transporter (DAT) in the striatum. (**A**): Representative images showing the TH^+^ neurons in the SNc area. (**B**): The number of DA neurons in the SNc was counted in each animal using unbiased Stereo Investigator system as described in Section 2. The number of DA neurons was significantly higher in the SNc of the control group (V + Sal) when compared to the MPTP-injected group (V + 1-methyl-4-phenyl 1,2,3,6 tetrahydropyridine (MPTP)). The ellagic acid (EA) treatment significantly prevented the DA neurons from the MPTP-induced neurodegeneration (*n* = 3–6 animals). (**C**): Representative images showing the immunoreactivity of dopamine transporter (DAT) in the striatum. The intensity of dopamine nerve terminals was significantly reduced in the striatum of MPTP-injected mice when compared with that in the control (V + Sal) group. Treatment with EA prior to MPTP injection shows significant attenuation of dopamine nerve terminals intensity. (**D**): DAT intensity was measured using NIH image J software and is presented in the graph. Values are expressed as a percentage of mean ± SEM relative to 100% control (*n* = 3–5 animals). The scale bar is 400 µm. Significance is denoted as * *p* < 0.05 and *** *p* < 0.001.

EA is present in many fruits and vegetables, especially pomegranates. It is credited with antioxidant and anti-inflammatory activities [7,8]. MPTP is a neurotoxin that selectively degenerates dopamine neurons and has been used to model PD in rodents and primates. It induces dopaminergic neurodegeneration due to the accumulation of oxidative stress and activation of neuroinflammation. Therefore, we first evaluated the neuroprotective efficacy of EA by evaluating the loss of TH-positive dopamine (DA) neurons in the SNc using unbiased stereological techniques and optical density of striatal dopamine fibers—the nerve terminal of DA neurons. MPTP administration caused a significant death of DA neurons in the SNc area and a decrease in density of striatal DA fibers of mice when compared to vehicle-injected control mice. However, treatment of EA prior to MPTP injection protected DA neurons in SNc and striatal DA fibers (*p* < 0.05) from the MPTP-induced toxicity when compared with MPTP-injected group animals. Moreover, EA alone did not cause any harmful effect on DA neurons in SNc and or its nerve terminal in the striatum (Figure 1). Taken together, this result suggests that EA treatment is beneficial, as it provides neuroprotection against MPTP in the SNc and striatum.

### 3.2. Effect of EA on Lipid Peroxidation and Glutathione Level in the Striatum Tissues

**Figure 2:**
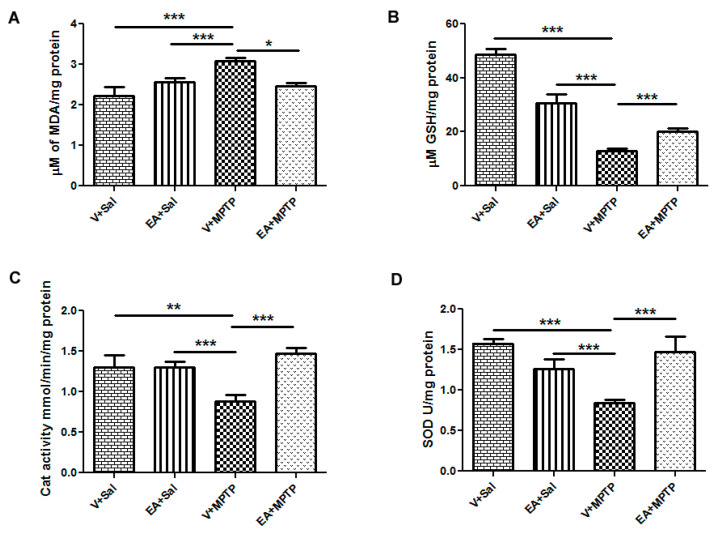
Estimation of malondialdehyde (MDA), glutathione (GSH), catalase, and superoxide dismutase (SOD) in the striatum tissue of different experimental groups. The MPTP injection increased the lipid peroxidation product, malondialdehyde (MDA) (**A**) and decreased the total glutathione (GSH) level (**B**) in the striatum tissue of mice relative to the control (Vehicle + Saline) group. MPTP injections also significantly decreased the activity of catalase (**C**) and SOD (**D**). Ellagic acid (EA) treatment prior to MPTP significantly increased the activities of the antioxidant enzymes SOD and catalase. It significantly reduced the level of MDA and increased the level of total GSH. Values are expressed as mean ± SEM (*n* = 4–5). Significance is denoted as * *p* < 0.05, ** *p* < 0.01 and *** *p* < 0.001.

EA has been credited with antioxidant and anti-inflammatory properties as mentioned. It has been shown to be effective treating against neurodegenerative diseases. Thus, we investigated whether the neuroprotective effect of EA against MPTP is mediated through its antioxidant and anti-inflammatory effect. As expected and shown before [21], mice administered with MPTP showed a significant increase in lipid peroxidation product, MDA, as compared to the control group (Vehicle + Saline). Concurrently, MPTP administration caused a significant (*p* < 0.01) decrease in GSH levels when compared to the control group. Since EA exhibits antioxidant activity, we investigated whether it has any role in the inhibition of MDA generation and normalizes the GSH level. We found that mice pretreated with EA before MPTP injection showed a significant reduction in MDA levels and increased GSH levels when compared to the MPTP group (Figure 2A,B and Table 1). However, mice treated with only EA did not show any significant alteration in the level of MDA. However, we observed a slight decrease in the GSH level when compared to control animals. The effect of EA on GSH is not clear at the moment; further study is required to explain it.

### 3.3. Effect of EA on Antioxidant Enzymes Activity in the Striatum Tissues

Next, we measured the activity of two antioxidant enzymes, CAT and SOD, which decreased significantly in MPTP-injected animals in comparison with control animals. However, treatment with EA significantly increased the activity of CAT and SOD when compared to the MPTP-treated group. We did not observe any significant changes in the activity of CAT between the controls and controls injected with only EA (Figure 2C and Table 1). However, EA-only-treated animals showed a slight decrease in SOD levels when compared to the control group (Figure 2D and Table 1). The reason that EA causes low SOD levels is currently unknown. Further study may be necessary to examine its effect on SOD activity.

### 3.4. Effect of EA on the Induction of Glial Cells Activation in the Striatum

**Figure 3:**
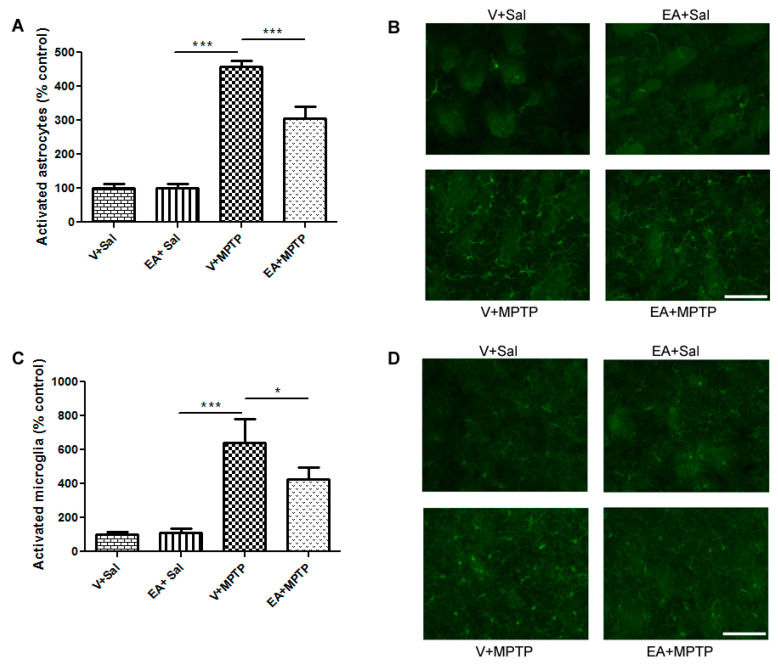
Immunofluorescence staining to detect the expression of activated glial fibrillary acidic protein (GFAP)-positive astrocytes (green) and ionized calcium-binding adaptor molecule-1 (Iba-1)-positive microglia (green) in the striatum of different groups of mice. Significantly higher levels of activated GFAP-positive astrocytes (**A**) and Iba-1-positive microglia (**C**) were found in the MPTP-treated mice relative to control mice (Vehicle + Saline). EA administration prior to MPTP injection in mice led to significantly lower numbers of activated GFAP and Iba-1 than MPTP-challenged mice. Quantitative analysis of activated astrocytes (**B**) and microglia (**D**) revealed a significant increase in the number of activated astrocytes and microglia in the mice injected with MPTP compared with the control mice. EA administration significantly reduced the number of activated astrocytes and microglia in the EA + MPTP group mice relative to the Vehicle + MPTP mice. Values are expressed as percentage of mean ± SEM (*n* = 3–6). The scale bar is 75 µm. Significance is denoted as * *p* < 0.05 and *** *p* < 0.001.

Immunofluorescence staining of GFAP and Iba-1 was carried out using striatum region similar to biochemical assay to assess the morphological changes seen in astrocytes and microglia upon administration of MPTP, respectively. It has been reported that activated GFAP and Iba-1 can be considered a marker of the inflammatory process. In this study, we observed significantly higher numbers of activated GFAP and Iba-1, as characterized by the distinct morphology in the MPTP-challenged mice when compared to control mice (Figure 3B,D). However, EA treatment prior to MPTP-challenged mice showed a comparatively lower immunofluorescence reactivity and decreased number of activated GFAP and Iba-1 astrocytes and microglia. The number of activated GFAP and Iba-1-positive astrocytes and microglia in the mice of different experimental groups was also counted. It is presented in Figure 3A,C. The administration of MPTP showed a significantly higher number of activated astrocytes and microglia compared to the control animals. Interestingly, treatment with EA prior to MPTP-challenged animals significantly reduced the number of activated astrocytes and microglia compared with MPTP-injected animals (Figure 3). In contrast, EA alone had no effect on either GFAP or Iba-1 morphology. These observations clearly demonstrate that EA inhibits the activation of microglia and astrocytes.

### 3.5. Effect of EA on the Induction of Pro-Inflammatory Cytokines in the Striatum Tissues

**Figure 4:**
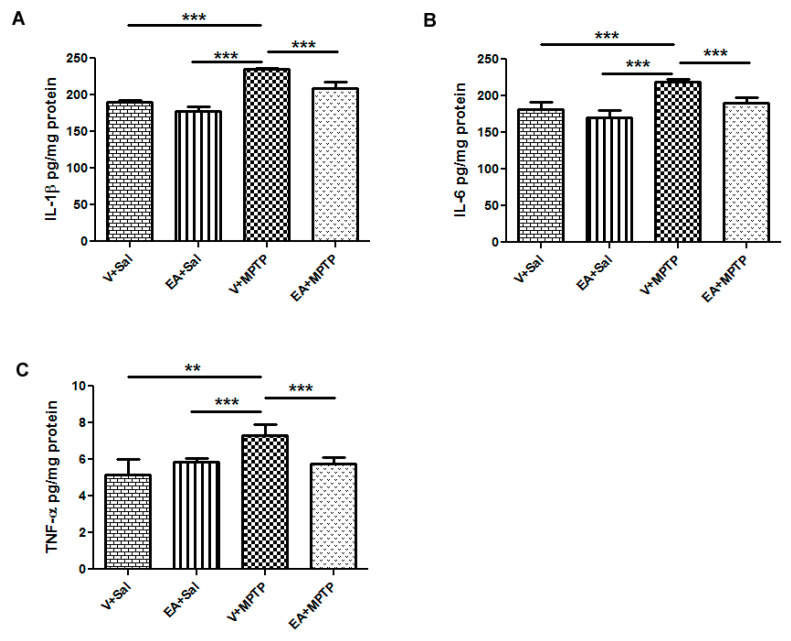
Determination of interleukin-1β (IL-1β), IL-6, and tumor necrosis factor-alpha (TNF-α) in the striatum tissue of different experimental groups animals using enzyme-linked immunosorbent assay (ELISA). MPTP treatment significantly increased the levels of IL-1β, IL-6, and TNF-α. EA treatment prior to MPTP injections significantly reduced the level of IL-1β (**A**), IL-6 (**B**), and TNF-α (**C**) when compared with MPTP-treated animals. Values are expressed as mean ± SEM (*n* = 4–5). Significance is denoted as ** *p* < 0.01 and *** *p* < 0.001.

We measured the concentration of pro-inflammatory cytokines IL-1β, IL-6, and TNF-α in the striatum tissues to determine the role of neuroinflammation in the MPTP model. We also examined how neuroinflammatory cytokines behave in the presence of EA. We found that MPTP administration induces significant increases in the level of pro-inflammatory cytokines such as IL-1β, IL-6, and TNF-α when compared with the control group. However, EA treatment prior to the MPTP injection significantly decreased the level of elevated pro-inflammatory cytokines in MPTP-challenged animals when compared with the MPTP group. Mice treated only with EA did not show any increase in the levels of pro-inflammatory cytokines when compared to control group animals (Figure 4 and Table 1). This result suggests that EA significantly prevented the increase of inflammatory cytokines, as expected.

### 3.6. Effect of EA on Inflammatory Mediators; Striatal COX-2 and iNOS Expression

**Figure 5:**
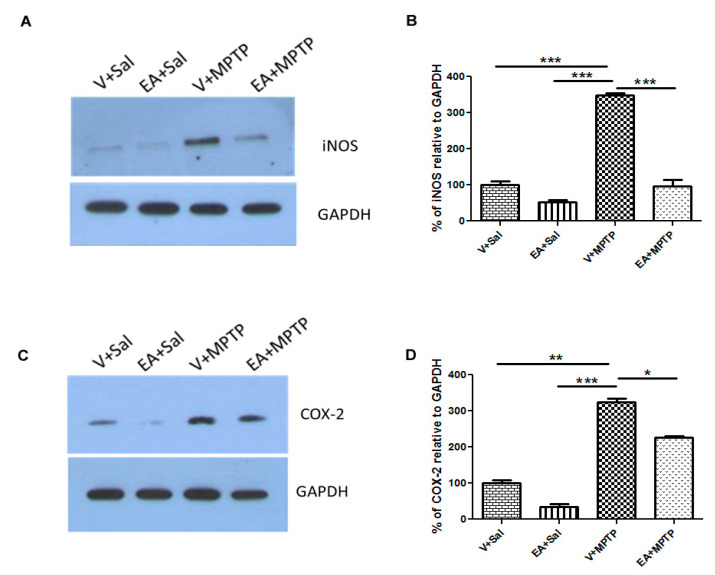
Expression of striatal inducible nitric oxide synthase (iNOS) and cyclooxygenase-2 (COX-2), as measured by Western blot analysis (**A**,**C**). A significant increase in iNOS was observed in the MPTP group relative to the control (Vehicle + Saline) group. Ellagic acid (EA) treatment prior to MPTP injection significantly decreased the expression of iNOS relative to the MPTP group (**A**,**B**). Similarly, COX-2 expression was increased significantly in MPTP group relative to the control (Vehicle + Saline) group. EA treatment decreased the COX-2 expression relative to the MPTP group (**C**,**D**). Values are expressed as percentage of mean ± SEM relative to control group (Vehicle + Saline). Significance is denoted as * *p* < 0.05, ** *p* < 0.01 and *** *p* < 0.001.

COX-2 and iNOS are crucial inflammatory mediators and execute many adverse effect cells or tissues. We assessed the expression of COX-2 and iNOS using Western blot analysis of the samples of striatal tissue lysates. The expression level of COX-2 was increased in MPTP-challenged mice compared with control mice, as reported earlier [21]. However, treatment with EA prior to the MPTP injection significantly decreased the elevated level of COX-2 compared with the MPTP group animals (Figure 5). Similarly, we observed an increase in iNOS expression in MPTP-injected animals when compared with the control animals. However, following treatment with EA prior to MPTP administration, a significant reduction in the level of iNOS was detected. Mice injected with EA only did not exhibit any alteration in the expression level of COX-2 and iNOS.

## 4. Discussion

PD is the second-most common neurodegenerative disorder after Alzheimer’s disease. There is no cure for this progressive and chronic disorder that can prevent neuronal death in the SNc area of the brain. The current medication can only improve the motor symptoms of the disease. Efforts have been made to develop new therapies to prevent neuronal death. Thus, the present study was undertaken to evaluate the neuroprotective effect of ellagic acid in an MPTP model of PD. Our data demonstrate that EA prevents MPTP-induced oxidative stress markers, such as a decreased MDA level and increased total GSH, catalase, and SOD activity. We also found that EA significantly prevents the activation of proinflammatory cytokines and their mediators. These data suggest that the neuroprotective effect of EA acts by (i) inhibiting oxidative stress, (ii) increasing the antioxidant enzymes/peptide, and (iii) preventing the activation of inflammatory cytokines and their mediators.

Ellagic acid is mostly abundant in fruits and vegetables. It possesses antioxidant properties due to the presence of a hydrophilic moiety with four hydroxyl groups in its chemical structure. It has been shown that EA can improve motor impairment in MFB-lesioned rats induced by 6-OHDA [17,18,19,22]. It is already known that 6-OHDA can cause significant neurodegeneration in the SNc area. It is speculated that EA can rescue neurons from oxidative stress induced by 6-OHDA. However, Baluchnejadmojarad et al. did not provide clear evidence that this effect was due to the preservation of dopamine pathways in the SNc and striatum area. Thus, from that study it was not clear whether the EA-mediated motor behavior improvement was due to the preservation of SNc neurons. Moreover, the 6-OHDA model only can be used to test motor behavior. EA has also been shown to protect DA neurons from rotenone-induced neurotoxicity through activation of Nrf2 signaling [15]. It is important to note that, although rotenone been used to model PD, it is not specific for DA neurons. On the other hand, the MPTP model has been considered one of the most suitable models to test the efficacy of potential compounds. It can recapitulate the pathological features of PD. Therefore, we were interested to examine the neuroprotective effect of EA in an in vivo mouse model of MPTP-induced PD. In the current study, we used a 25 mg/kg body weight single dose of MPTP for 5 consecutive days to induce selective dopaminergic neuronal death in the SNc area, similar to that seen in human PD patients. The MPTP injection in mice induces oxidative stress and neuroinflammation, which are considered key factors causing death of dopamine neurons in the SNc area. Studies have shown that experimental animals, such as mice and monkeys, that received MPTP exhibit clinical and pathological features of PD, such as loss of TH-positive dopaminergic neurons in the SNc area by oxidative damage, inflammation, glial activation, and/or motor dysfunction [23,24,25,26,27]. To test the therapeutic potential of EA in this PD model is worthwhile considering the chronic, progressive, and sporadic nature of PD. The dose of EA used in this study was chosen based on our preliminary experiments as well as previous published studies.

The brain sections of mice upon immunohistochemical analysis revealed significant degeneration of the dopaminergic neurons in the SNc area following MPTP challenge in agreement with numerous previous studies including our own [21,28,29]. The cell bodies of DA neurons in the SNc project their nerve terminal to the striatum; thus, the degeneration of DA neurons in the SNc area results in the retraction of the DA nerve terminal in the striatum region. As expected, we observed that MPTP administration significantly reduced the number of TH immunoreactive neurons in the SNc area as well as the DA nerve terminal density in the striatum (Figure 1). We found that EA treatment prior to MPTP administration in mice afforded significant protection to DA neurons and preserved the integrity of DA nerve terminals. This suggests that EA is beneficial for DA neurons as it prevents the neurons from MPTP-induced toxicity.

Oxidative stress plays an important role in causing the death of dopamine neurons. Additionally, biochemical analyses of postmortem PD brains show enhancements of oxidative stress due to perturbations of the mitochondrial electron transport chain. This may produce an imbalance between the toxic oxidant and the cellular antioxidant defense system. The toxic oxidants are reactive oxygen species (ROS) such as superoxide radical (O_2_), hydroxyl radical (HO), hydrogen peroxide (H_2_O_2_), nitric oxide (NO), and peroxynitrite (ONOO). The endogenous cellular antioxidant defense system comprises of enzymes (SOD and CAT) and nonenzymatic tripeptide (GSH). The decrease in the activities of endogenous antioxidant defense system, such as GSH, SOD, and CAT, has been well documented in PD brains [30]. In this study, following MPTP exposure, a significant reduction in SOD and CAT activities in the striatum tissues was recorded. However, administration of EA prior to MPTP caused a significant improvement in the SOD and CAT activities, clearly demonstrating the antioxidant effect of EA. Moreover, we observed a significant increase in the level of MDA and a reduction in the GSH contents in the brain tissues after MPTP administration. MDA is the product of lipid peroxidation and is used as a marker for oxidative stress. Higher levels of MDA are found in MPTP-challenged mice. Additionally, MPTP induces GSH depletion and reduces GSH levels, possible due to a decline in intracellular NAD^+^ and ATP stores, which are necessary for GSH anabolism, release, and consequent hydrolysis. This result clearly demonstrates the imbalance between the antioxidant defense system and oxidative stress caused by MPTP. However, EA treatment prior to MPTP administration significantly increased the GSH level and decreased the MDA level, again suggesting the antioxidant function of EA. In several studies, EA has been widely reported to exert free radical scavenging and potent antioxidant activity by scavenging ROS and restoring the activity of cellular antioxidant enzymes [31,32,33,34,35].

Oxidative stress and inflammation are interrelated pathological events. Inappropriate glial activation contributes to pathological outcomes, such as neurodegenerative disease [36,37], and the activation of glial cells after MPTP exposure has been well documented [27]. The activated microglia release several pro-inflammatory cytokines, such as IL-1β, IL-6, and TNF-α, in the surrounding brain tissue, which are accompanied by persistent activation of microglia. To examine the possible effects of EA on the inflammatory pathways, levels of various inflammatory molecules and pro-inflammatory cytokines were assessed. In this study, following MPTP administration, we found an increased level of pro-inflammatory cytokines, such as IL-1β, IL-6, and TNF-α, in the striatum samples, which is in accordance with findings of previous studies [21]. EA treatment prior to MPTP exposure significantly reduced the level of IL-1β, IL-6, and TNF-α induction along with microglial activation. This result suggests that EA might counteract the activation process of microglia, thereby controlling the levels of IL-1β, IL-6, and TNF-α. Indeed, several studies have shown ellagic acid as a potential anti-inflammatory compound for many diseases [38,39,40]. The inhibition of glial activation and subsequent production of pro-inflammatory mediators may offer prospective therapeutic value for the treatment of neuroinflammation-related neurodegenerative disorders including PD. Consequently, we examined the activation profile of microglia and astrocytes upon MPTP exposure and the role of ellagic acid in this model. We observed increased activation of Iba-1 and GFAP, which are considered markers of glial activation and the inflammatory process. However, mice treated with EA showed a significant reduction in the activation of microglia and astrocytes, as evidenced by the reduction in the expression of Iba-1 and GFAP, respectively.

In this study, we observed that EA prevents the release of endogenous inflammatory mediators (e.g., COX-2, iNOS, and cytokines) in striatum tissue of mice in an MPTP-induced PD model. The anti-inflammatory effect of EA was further confirmed by examining its inhibitory effect on COX-2 and iNOS activity. It is well reported that iNOS and COX-2 levels increase in PD patients. Elevated levels of iNOS amplify the inflammatory response and lead to the production of more cytokines. On the other hand, COX-2 induction enhances the cytotoxic effect by generating pro-inflammatory prostaglandins as well as ROS. We also observed a remarkable increase in COX-2 and iNOS expression following MPTP injection, which is consistent with earlier reports. However, the administration of EA to MPTP-challenged mice reduced the increase of COX-2 and iNOS levels, which can be plausibly explained by its strong anti-inflammatory activity. In previous studies, EA was shown to exert potent anti-inflammatory effects by inhibiting the NF-kB pathway [41].

## 5. Conclusions

Parkinson’s disease is the second most common type of neurodegenerative disorder after Alzheimer’s disease. The current available treatment can only improve the symptoms but is unable to prevent neurodegeneration. In the search for new beneficial compounds, our study demonstrates that EA protects dopamine neurons very effectively in an MPTP-induced mouse model of PD by inhibiting the oxidative stress and inflammation-related toxicity. As a naturally occurring compound, EA also has various beneficial biological effects on different organ systems through the regulation of oxidative stress and inflammation. Thus, EA may represent a more reasonable natural agent that has potential therapeutic value for future clinical study. However, the exact molecular mechanism by which EA acts requires further investigation.

## Figures and Tables

**Table 1:** Oxidative and inflammatory indicators of different experimental groups.

Groups of Animal	Oxidative Indicators				Inflammatory Indicators		
	SOD U/mg Protein	Catalase mmol/mg Protein	GSH µM/mg Protein	MDA µM/mg Protein	IL-1β pg/mg Protein	IL-6pg/mg Protein	TNF-αpg/mg Protein
Vehicle + saline	1.58 ± 0.06	1.31 ± 0.14	46.54 ± 2.06	2.22 ± 0.23	190.63 ± 2.45	180.97 ± 9.90	5.18 ± 0.82
EA + saline	1.26 ± 0.13	1.30 ± 0.07	32.44 ± 4.56	2.57 ± 0.10	177.71 ± 6.66	169.72 ± 11.03	5.84 ± 0.19
Vehicle + MPTP	0.84 ± 0.04	0.88 ± 0.08	15.54 ± 0.97	3.08 ± 0.12	234.61 ± 2.41	218.71 ± 3.89	7.31 ± 0.59
EA + MPTP	1.47 ± 0.19 ***	1.47 ± 0.06 ***	18.17 ± 1.59 ***	2.45 ± 0.10 *	209.14 ± 8.49 ***	189.93 ± 7.16 ***	5.75 ± 0.38 ***

Values are expressed in mean ± SEM of 4–5 animals in each group. Statistics: * p < 0.05, *** p < 0.001 indicate the comparison with the Vehicle + MPTP group.
